# Hepatocyte growth factor-like protein is required for prostate tumor growth in the TRAMP mouse model

**DOI:** 10.18632/oncotarget.2139

**Published:** 2014-06-26

**Authors:** Juozas Vasiliauskas, Madison A. Nashu, Peterson Pathrose, Sandra L. Starnes, Susan E. Waltz

**Affiliations:** ^1^ Departments of Cancer Biology, Cincinnati Veterans Affairs Medical Center, Cincinnati, Ohio; ^2^ Department of Surgery, University of Cincinnati College of Medicine, Cincinnati Veterans Affairs Medical Center, Cincinnati, Ohio; ^3^ Research Service, Cincinnati Veterans Affairs Medical Center, Cincinnati, Ohio

**Keywords:** Ron receptor, hepatocyte growth factor-like protein, prostate cancer, Mst1R

## Abstract

The Ron receptor is deregulated in a variety of cancers. Hepatocyte growth factor-like protein (HGFL) is the ligand for Ron and is constitutively secreted from hepatocytes into the circulation. While a few recent reports have emerged analyzing ectopic HGFL overexpression in cancer cells, no studies have examined the effect of host-produced HGFL in tumorigenesis. To examine HGFL function in prostate cancer, the TRAMP mouse model, which is predisposed to develop prostate tumors, was utilized. Prostate tumors from TRAMP mice exhibit elevated levels of HGFL, which correlated with upregulation in human prostate cancer. To directly implicate HGFL in prostate tumorigenesis, TRAMP mice deficient in HGFL (HGFL-/-TRAMP+) were generated. HGFL-/- TRAMP+ mice developed significantly smaller prostate tumors compared to controls. Analysis of HGFL-/- tumors revealed reduced tumor vascularization. No differences in cancer cell proliferation were detected between HGFL-/- TRAMP+ and HGFL+/+ TRAMP+ mice. However, a significant increase in cancer cell death was detected in HGFL-/- TRAMP+ prostates which correlated with decreased pro-survival targets. *In vitro* analysis demonstrated robust STAT3 activation resulting in Bcl2-dependent survival following treatment of prostate cancer cells with HGFL. These data document a novel function for endogenous HGFL in prostate cancer by imparting a critical survival signal to tumor cells.

## INTRODUCTION

Prostate cancer persists as one of the highest incidences of cancer in men in the United States. According to American Cancer Society, there were more than 240,000 new prostate cancer cases in 2012 alone with more than 28,000 estimated deaths [1]. Receptor tyrosine kinases have been implicated in the development and progression of various cancers including prostate cancer [reviewed in [2]]. One of these receptors, the Ron receptor tyrosine kinase, has been implicated as an oncogene in numerous reports [3-7], and is emerging as a cancer therapeutic target [6]. While the use of selective inhibitors and monoclonal antibodies against Ron have shown promise *in vitro and in vivo* inhibiting tumor growth, potential side effects and lack of complete inhibition [6, 8, 9] call for the development of new therapeutic approaches. Hepatocyte growth factor-like protein, HGFL, is the ligand for Ron receptor tyrosine kinase. HGFL is primarily produced by hepatocytes and is secreted into the bloodstream in an inactive pro-HGFL form [10] which when proteolytically cleaved binds to and activates Ron in an endocrine fashion at distant sites. Binding of HGFL to Ron results in the phosphorylation of tyrosine residues within the kinase domain of Ron [11]. Ron activation triggers a wide variety of downstream signaling cascades that are utilized in cellular proliferation, migration and cell scattering amongst others [10, 12]. Recently, we have shown that in murine models of breast and prostate cancer, mice deficient in Ron downstream signaling present with reduced tumor burden [13, 14]. Despite our knowledge of Ron, HGFL function in tumorigenesis has not been well studied. Of the few reports, ectopic overexpression of HGFL led to increased metastasis in models of breast cancer and small cell lung carcinoma [15, 16]. No studies however, have examined endogenous HGFL function and its importance in prostate cancer tumorigenesis. To elucidate the role of HGFL in prostate tumorigenesis *in vivo*, we generated TRAMP (Transgenic Adenocarcinoma of Mouse Prostate) mice that are deficient in HGFL (HGFL-/- TRAMP+). Prostates of male TRAMP mice develop hyperplastic regions by 10 weeks, and by 28 weeks of age 100% of mice develop prostate cancer [17]. We have previously utilized this mouse model of prostate cancer to show the functional importance of the Ron receptor tyrosine kinase in prostate tumorigenesis [13], however our findings did not clarify if the functional role of Ron in prostate cancer was dependent on ligand activation. In this report, we demonstrate that HGFL is a critical factor for the development of prostate tumorigenesis in this model through the promotion of the oncogenic activation of Ron and the induction of strong signals required for efficient prostate cancer cell survival.

## RESULTS

### HGFL is deregulated in human prostate cancer

**Figure 1:**
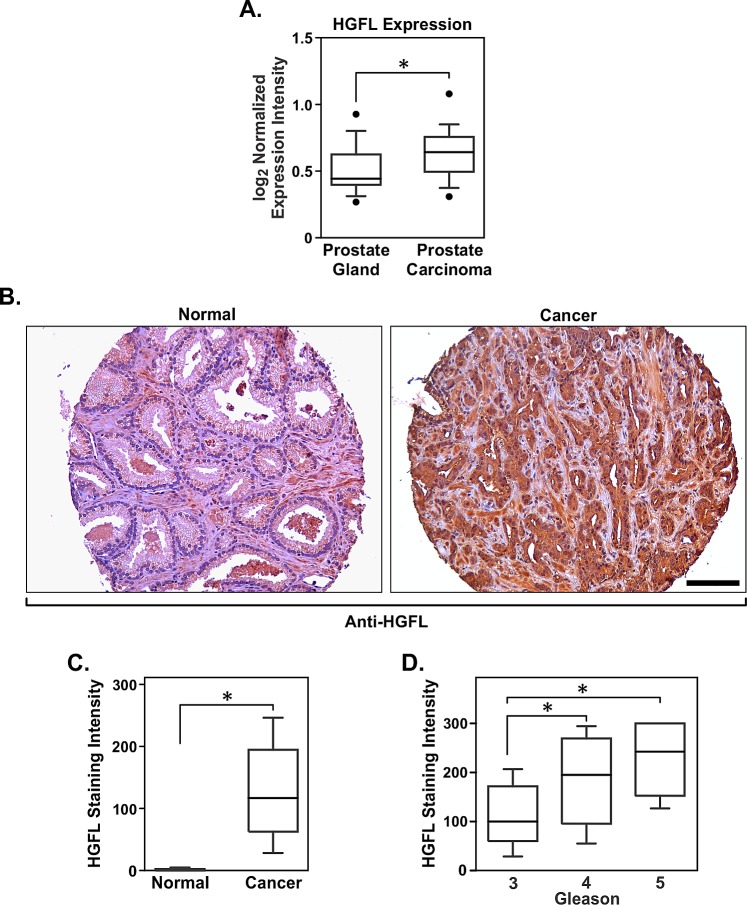
HGFL is expressed at the mRNA and protein levels in human prostate cancer A). Boxplot depicting the relative HGFL mRNA expression in clinical prostate samples. B). Immunohistochemical analysis showing HGFL upregulation in prostate cancer epithelial cells. Scale bar=100um. C). HGFL staining intensity scoring on human normal prostate (n=191) and prostate cancer specimens (n=188). Boxplot lists 25^th^ and 75^th^ percentiles along with the group median. Whiskers display 10^th^ and 90^th^ percentiles. D). Boxplot as in C displaying the association between HGFL staining intensity and Gleason score in prostate cancer. HGFL expression increases with disease progression (n=152 for Gleason 3, n=24 for Gleason 4 and n=9 for Gleason 5). *P<0.05.

Ectopic HGFL expression has been shown to increase the sites of metastatic involvement in a murine breast cancer mouse model [15] and its deregulation in conjunction with its receptor, Ron, was observed in human breast cancer patients. However, little is known about HGFL in prostate tumorigenesis. Oncomine microarray analyses (Figure 1A) demonstrate that HGFL mRNA is produced locally in the prostate with elevated expression observed in prostate carcinomas [18]. To delineate the cell type responsible for HGFL production within the prostate, human prostate cancer tissues were analyzed for HGFL expression by immunohistochemistry. While stromal staining for HGFL was weakly present, prostate cancer epithelial cells stained positive for HGFL expression. High levels of HGFL were observed in tumor tissue compared to relatively undetectable expression in normal, nondiseased, prostate tissue (Figure 1B and 1C). The majority of human prostate cancer samples exhibited HGFL staining with scores ranging from 0 to 300 and a median value of 117 compared to a range of 0-20 and a median value of 0 for normal tissues. Figure 1B shows representative HGFL staining in normal human prostate tissue and in prostate cancer. Of the 188 human prostate cancer samples examined, 184 exhibited staining for HGFL to various degrees. Moreover, an analysis of the clinical data associated with human prostate cancer tissue specimens revealed a strong association between HGFL staining and Gleason score (Figure 1D). Taken together, the data demonstrate HGFL expression within human prostate cancer cells.

### Prostates from HGFL deficient mice are phenotypically similar to prostates from wild-type (HGFL+/+) mice

**Figure 2:**
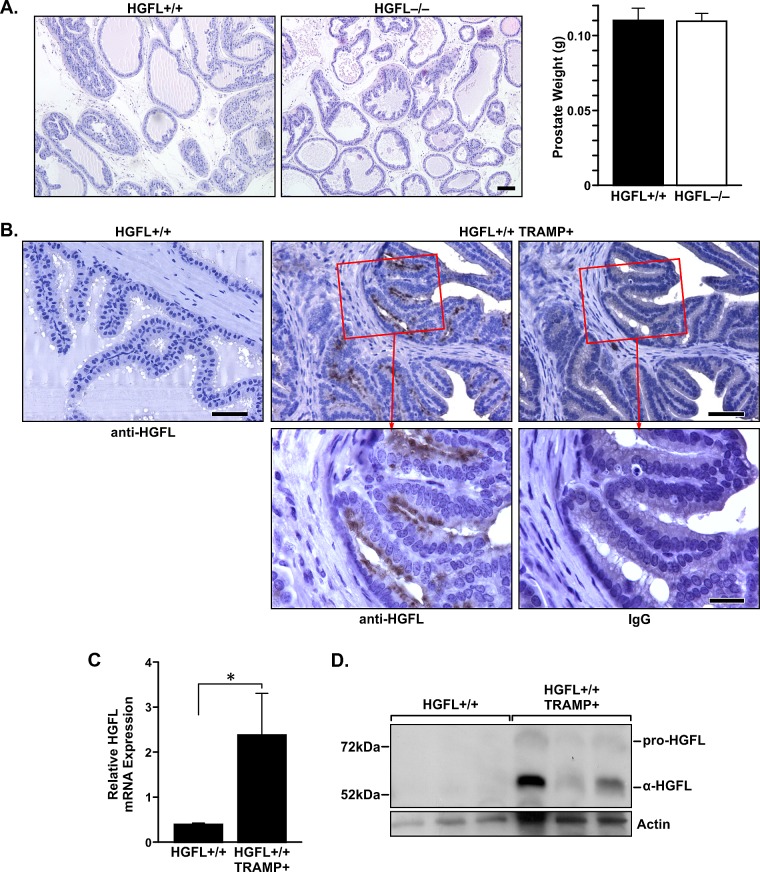
HGFL is expressed in murine prostate cancer A). Representative H&E staining of 30-week old HGFL+/+ and HGFL-/- prostate sections. Scale bar=100um. Bar graph listing prostate weights from HGFL proficient (n=6) and deficient (n=20) mice. B). Prostate tumor epithelial cells of HGFL+/+ TRAMP+ mice express HGFL as detected by immunohistochemical staining. Scale bar is 50um and insert scale bar is 20um. C). qRT-PCR showing HGFL mRNA expression in the prostates of HGFL+/+ TRAMP+ mice (n=8 mice) compared to prostates from HGFL+/+ mice (n=6). Data are expressed as the mean ± SE, *P<0.05. D). Total prostate lysates were prepared from 30-week old HGFL+/+ control and HGFL+/+ TRAMP+ mice and analyzed for HGFL expression by Western blot analysis. Actin is shown as a loading control.

Prostates from HGFL+/+ and HGFL-/- mice were analyzed by hematoxylin and eosin staining to determine if HGFL loss led to any phenotypic variation. While prostate pathology was overtly similar, slight abnormalities were detected in the prostates of 30-week old HGFL-/- animals compared to the controls which are summarized in Supplementary Figure S1. The prominent features of 30-week old HGFL-/- prostates were the presence of prominent nuclei and atypical cells which occurred in 91% of HGFL-/- prostates compared to 84% in HGFL+/+ prostates, with occasional disrupted gland profile. At 14 weeks of age, prostates from both groups were similar (data not shown). Despite the moderate increase in prostate pathology in the 30-week old HGFL-/- mice, prostate tumors did not develop in the HGFL-/- mice. Representative prostate tissue from HGFL+/+ and HGFL-/- mice and average prostate weights are depicted in Figure 2A.

### HGFL is expressed locally in TRAMP+ prostate tumors

To examine HGFL expression in the prostates of wild-type and TRAMP+ mice, 30-week old HGFL+/+ and HGFL+/+ TRAMP+ prostates were stained for HGFL expression by immunohistochemistry (Figure 2B). While no HGFL staining was detected in HGFL+/+ prostates, an increase in HGFL expression in prostate epithelial cells was detected in HGFL+/+ TRAMP+ prostates. Moreover, HGFL mRNA levels were analyzed by qRT-PCR and an increase in HGFL transcripts was detected within HGFL+/+ TRAMP+ mouse prostates compared to the prostates of wild-type counterparts (Figure 2C). Western blot analyses of HGFL expression on whole prostate lysates of HGFL+/+ TRAMP+ mice also show elevated levels of HGFL compared to HGFL+/+ mice (Figure 2D).

### HGFL loss dramatically reduces genitourinary complex and prostate tumor size in TRAMP+ mice

**Figure 3:**
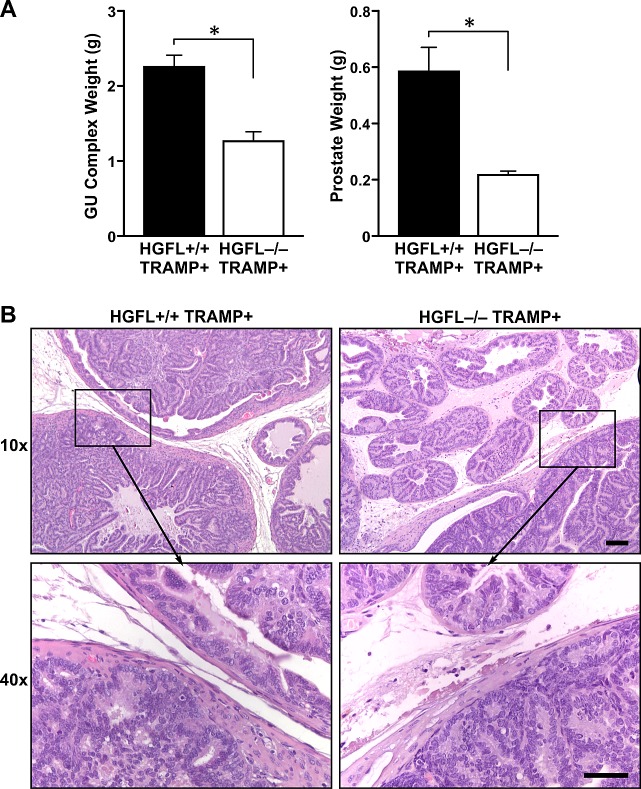
HGFL deficient TRAMP+ mice have reduced GU complex and prostate tumor size A). 30-week old HGFL-/- TRAMP+ mice (n=8) exhibit decreased GU complex and prostate tumor mass relative to HGFL+/+ TRAMP+ mice (n=12). Data are represented as the mean ± SE. *P<0.05. B). Representative histological analysis of HGFL+/+ TRAMP+ and HGFL-/- TRAMP+ prostates. H&E staining shows neoplastic tumor development in HGFL+/+ TRAMP+ and HGFL-/- TRAMP+ prostates. Scale bar is 100um and insert scale bar is 50um.

30-week old HGFL-/- TRAMP+ mice had significantly smaller genitourinary complex (GU) and prostate sizes compared to TRAMP+ mice proficient for HGFL (Figure 3A). The average size of 30-week old HGFL-/- TRAMP+ prostates was 0.21g with a GU complex weight of 1.26g whereas the average weight of HGFL+/+ TRAMP+ prostates was 0.58g with a GU complex weight of 2.26g. For comparison, the average size of HGFL+/+ prostate and GU complex were 0.13g and 0.58g, respectively, and were similar to the prostates of HGFL-/- animals (data not shown). Histologically, the prostate tumors were similar in TRAMP+ mice regardless of HGFL status. Both genotypes of mice exhibited enlarged prostates that varied from normal prostatic epithelial cell structures to well-differentiated adenocarcinoma as well as defined glandular architecture (Figure 3B). At the 30-week time point in our colony, no metastases were detected in the lungs, local lymph nodes, or livers of HGFL+/+ TRAMP+ or HGFL-/- TRAMP+ mice.

### HGFL loss does not alter Ron expression in the prostates of TRAMP+ mice

**Figure 4:**
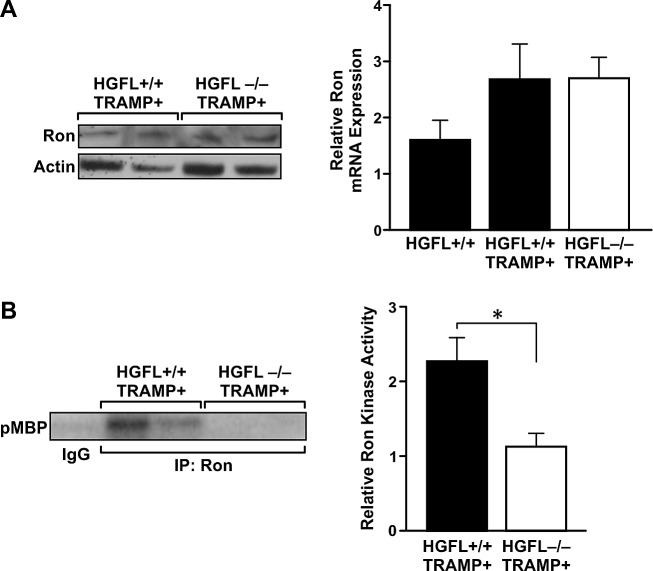
Decreased Ron activity in the prostates of HGFL-/- TRAMP+ animals A). Ron expression levels are similar in HGFL+/+ TRAMP+ and HGFL-/- TRAMP+ mice prostate tumors as judged by Western blot and qRT-PCR analyses. qRT-PCR analysis shows representative data from 2 independent experiments (n=4 mice per group). B). Ron kinase activity is decreased in prostate tumors from HGFL -/- TRAMP+ compared to HGFL+/+ TRAMP+ mice as depicted by kinase assays examining myelin basic protein (MBP) as a substrate. Graph shows combined data from 5 independent experiments (n=4 mice per group) ± SE normalized to Ron expression levels in A. *P<0.05.

To determine if Ron expression is attenuated in the absence of HGFL, 30-week prostates from HGFL-/- TRAMP+ and HGFL+/+ TRAMP+ mice were analyzed by Western blot and qRT-PCR analyses. Ron protein and transcript levels were unchanged in the prostates of HGFL-/- TRAMP+ animals compared to the prostates of HGFL+/+ TRAMP+ mice (Figure 4A). Interestingly, even though total levels of Ron were not altered, Ron isolated from the prostates of HGFL+/+ TRAMP+ animals exhibited significantly higher kinase activity compared to Ron from the prostates of HGFL-/- TRAMP+ animals (Figure 4B).

### Prostate tumors from animals lacking HGFL have similar proliferation rates, but exhibit increased cell death

**Figure 5:**
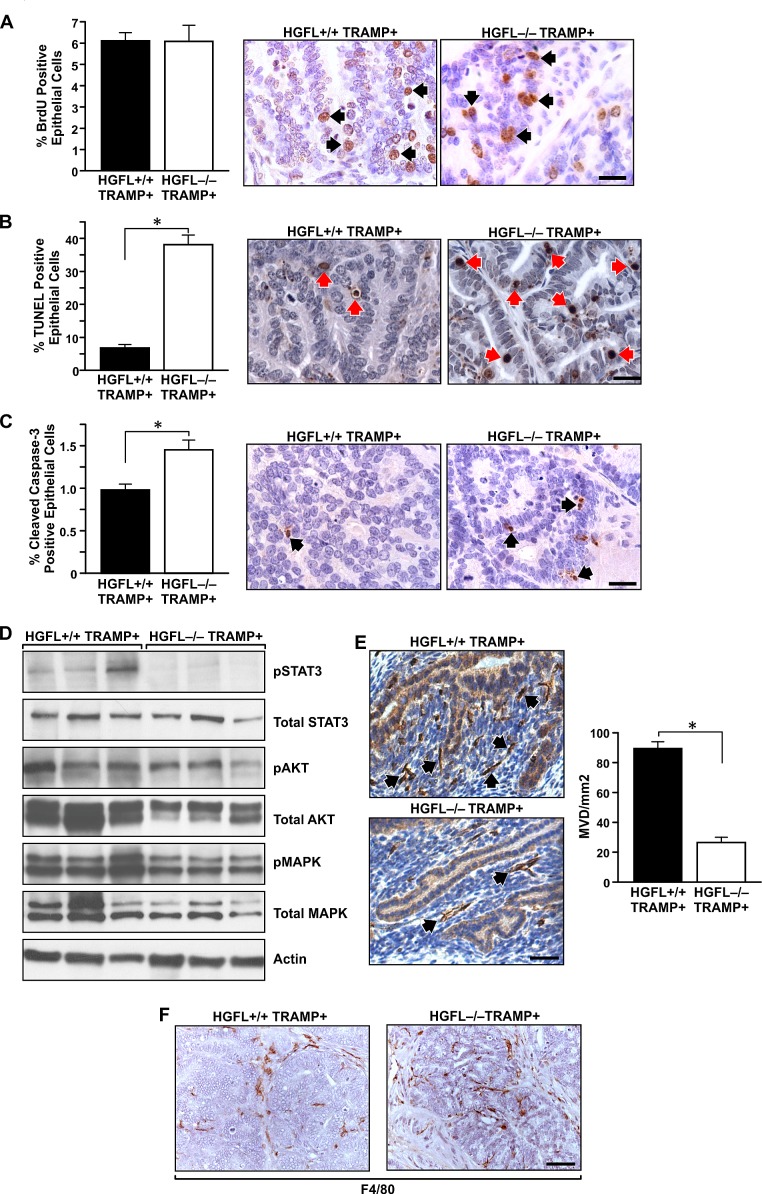
Prostate tumor characterization from HGFL+/+ TRAMP+ and HGFL-/- TRAMP+ mice A). BrdU immunostaining was performed on prostates from 30-week HGFL+/+ TRAMP+ and HGFL-/- TRAMP+ mice. Prostates from 30-week old HGFL+/+ TRAMP+ and HGFL-/- TRAMP+ mice did not exhibit significant differences in BrdU incorporation. Data are expressed as the mean ± SE. Three separate areas were counted from three independent specimens per group, and representative images are shown. Arrows depict a few of the positive cells stained for BrdU. Scale bar=20um. B). Detection of TUNEL-positive cells in prostate tissue of HGFL+/+ TRAMP+ and HGFL-/- TRAMP+ mice at 30-weeks of age. Prostates from 30-week old HGFL+/+ TRAMP+ and HGFL-/- TRAMP+ mice stained significantly different for TUNEL. Data are expressed as the mean ± SE. *P<0.05. Three separate areas were counted from four independent specimens per group, and representative images are shown. Arrows depict a few of the positive cells stained for TUNEL. Scale bar=20um. C). Cleaved Caspase-3 immunostaining was performed on prostates from 30-week HGFL+/+ TRAMP+ and HGFL-/- TRAMP+ mice. Prostates from 30-week old HGFL-/- TRAMP+ mice had significantly higher Cleaved Caspase-3 staining. Data are expressed as the percent positive prostate epithelial cells ± SE. *P<0.05. Four separate areas were counted from three independent specimens per group, and representative images are shown. Arrows depict a few of the positive cells stained for Cleaved Caspase-3. Scale bar=20um. D). Western blot analyses of whole prostate tumor lysates from HGFL+/+ TRAMP+ and HGFL-/- TRAMP+ mice for downstream Ron signaling targets. E). HGFL-/- TRAMP+ mice have decreased vascularization as determined by CD31 staining compared to HGFL+/+ TRAMP+ mice. Bar graphs depict mean vessel density per area with n=3 mice per each group and 3 random fields per mouse. Representative images are shown. Data are expressed as the mean ± SE. *P<0.05. Scale bar=20um. F). F4/80 immunostaining was performed on prostates from 30-week HGFL+/+ TRAMP+ and HGFL-/- TRAMP+ mice. F4/80-positive cells infiltrated prostate tumors from HGFL-/- TRAMP+ mice whereas F4/80-positive cells localized on the tumor periphery in prostate tumors from HGFL+/+ TRAMP+ mice.

Since 30-week old prostates from HGFL-/- TRAMP+ animals displayed significantly smaller prostate tumor size compared to HGFL+/+ TRAMP+ mice, rates of cellular proliferation and death between the two groups were determined. No appreciable differences in prostate epithelial cell proliferation were detected between HGFL+/+ TRAMP+ and HGFL-/- TRAMP+ mice as determined by bromodeoxyuridine (BrdU) incorporation (Figure 5A). However, a significant increase in the number of TdT-mediated dUTP nick end labeling (TUNEL) positive cells was detected in HGFL-/- TRAMP+ prostate tumors (Figure 5B). This finding correlated with previous findings wherein prostate tumors from Ron tyrosine kinase deficient (TK-/-) TRAMP+ mice also exhibited reduced survival [13]. To further analyze prostate epithelial cell survival, Cleaved-Caspase 3 immunohistochemical staining was performed on prostate tumors from mice proficient and deficient for HGFL. Prostate tumors from HGFL-/- TRAMP+ mice had a 1.5-fold increase in Cleaved-Caspase 3 positive epithelial cell staining compared to tumors from HGFL+/+ TRAMP+ mice (Figure 5C). Upon further analyses of downstream signaling targets of HGFL-induced Ron activation, decreases in the activation of AKT and MAPK were observed in prostate tumors of HGFL-/- TRAMP+ mice compared to HGFL+/+ TRAMP+ mice. We previously showed that prostate tumors from TRAMP+ mice deficient for Ron signaling exhibited reduced levels of nuclear NF-κB, suggesting reduced NF-κB activity [13]. Surprisingly, no appreciable differences in NF-κB signaling were detected between prostate tumors from HGFL+/+ TRAMP+ and HGFL-/- TRAMP+ mice (data not shown). Upon further analysis, the most striking alteration in downstream signaling was in the activation of STAT3 between genotypes. HGFL+/+ TRAMP+ prostate tumors displayed prominent STAT3 phosphorylation, while prostate tumors from HGFL-/- TRAMP+ mice exhibited no detectable STAT3 activation (Figure 5D).

### HGFL promotes angiogenesis and regulates macrophage invasion into the prostate tumors of TRAMP+ mice

Given that our previous studies demonstrated that loss of Ron was important for the formation of the prostate tumor vasculature in TRAMP+ mice [13], we next assessed whether prostate tumor vessel formation was ligand dependent. Prostate tumor sections from HGFL+/+ TRAMP+ and HGFL-/- TRAMP+ mice were immunostained for CD31 expression. Prostate tumors form HGFL-/- TRAMP+ mice showed a significant decrease in the number of microvessels per unit area as compared to the tumors from HGFL+/+ TRAMP+ mice (Figure 5E). These data suggest that the absence of ligand-dependent Ron activation is responsible for the decrease in vessel density in the tumors from HGFL-/- TRAMP+ mice and may be causal in increasing tumor cell death.

Since Ron has previously been shown to regulate macrophage migration and activation [19-21], macrophage recruitment into the prostates of HGFL+/+ TRAMP+ and HGFL-/- TRAMP+ mice was examined. Interestingly, the majority of F4/80-positive macrophages were localized at the tumor periphery in the prostates of HGFL+/+ TRAMP+ mice whereas prostate tumors from HGFL-/- TRAMP+ mice displayed F4/80-positive cells throughout the tumor proper (Figure 5F). This alteration in macrophages may also contribute to the enhanced tumor cell death observed in the prostates of HGFL-/- TRAMP+ mice.

### HGFL promotes prostate cancer cell survival through Ron-dependent regulation of STAT3 and Bcl2

**Figure 6:**
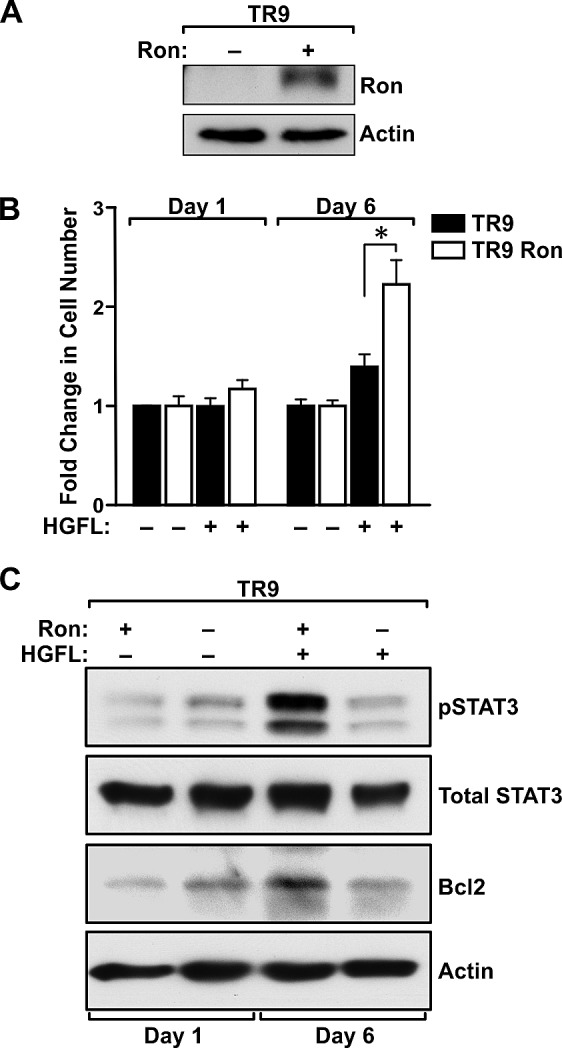
HGFL promotes survival of Ron expressing prostate cancer cells and induces STAT3 activation and Bcl2 expression under detached conditions A). Western blot depicting lentiviral transduction of a Ron cDNA into prostate cancer epithelial cells (TR9) derived from prostate tumors from TRAMP+ mice deficient for the Ron receptor. B). MTT assays demonstrate a survival advantage for TR9 Ron prostate cancer cells under serum-free conditions in presence of HGFL. Bar graph depicts the fold change in cell number compared to untreated cells and is representative of data from three independent experiments performed in duplicate. Data are expressed as the mean ± SE, *P<0.05. C). Western blot analysis of serum-starved TR9 and TR9 Ron cells in presence and absence of HGFL. A significant increase in pSTAT3 and Bcl2 levels was observed in TR9 Ron cells upon treatment with recombinant HGFL at day 6.

Given the dramatic increase in the survival of prostate tumor cells in HGFL+/+ TRAMP+ mice compared to those without HGFL, we next sought to examine whether STAT3 activation acts as a survival mechanism downstream of Ron signaling in prostate cancer cells *ex vivo*. To accomplish this and to examine the specificity of HGFL for Ron activation, prostate cancer cells were generated from TRAMP+ prostate tumors lacking Ron signaling (referred to as TR9 cells). TR9 cells were subsequently lentiviraly transduced to re-express mouse Ron (referred to as TR9 Ron cells) (Figure 6A). Since HGFL has previously been shown to prevent anoikis [22], the survival of TR9 and TR9 Ron cells was examined under detached conditions in the presence and absence of HGFL. Cell viability was measured at 1 and 6 days. As depicted in Figure 6B, HGFL treatment promoted the survival of TR9 cells only following reconstitution with Ron. In an examination of downstream signaling events associated with increased prostate cancer survival, a dramatic increase in STAT3 phosphorylation was observed in Ron expressing cells in presence of HGFL (Figure 6C). An increase in Bcl2 levels in TR9 Ron cells was also observed with HGFL treatment and is consistent with studies demonstrating STAT3 induction of Bcl2 [23, 24].

**Figure 7:**
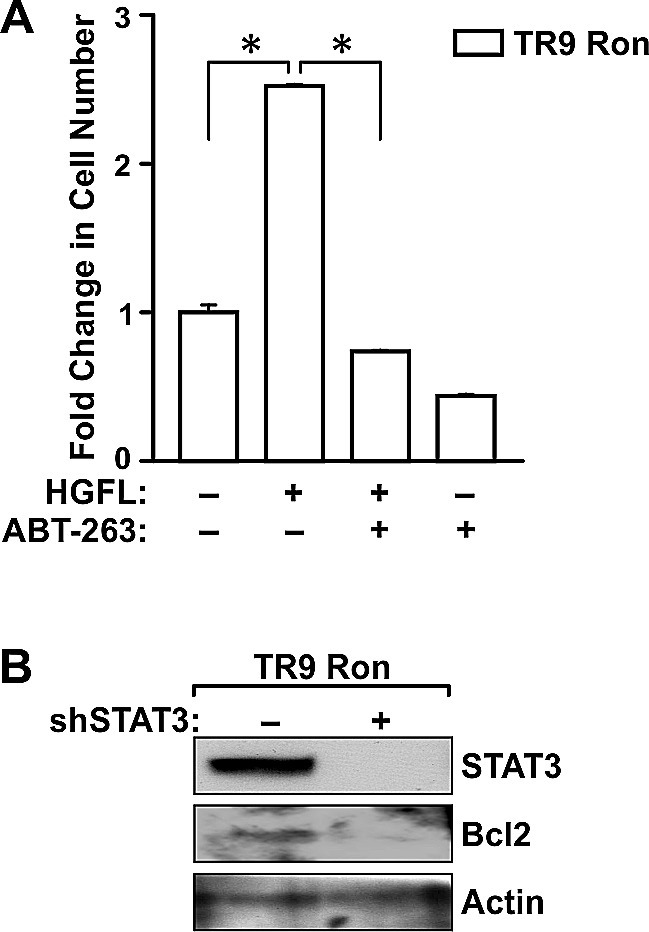
Bcl2 and STAT3 are required for HGFL-induced prostate cancer cell survival A). The survival of TR9 Ron cells was examined following 6 days under detached conditions. Cells were treated in the presence or absence of the Bcl2 inhibitor, ABT-263, with or without HGFL treatment. Cell viability was measured by MTT assays. The histogram depicts the fold change in cell number compared to untreated cells and is representative of data from two independent experiments performed in duplicate. Data are expressed as the mean ± SE, *P<0.05. B). Western blot analyses of TR9 Ron cells following transduction with a shRNA targeting STAT3. STAT3 loss led to diminished Bcl2 levels.

To examine the involvement of Bcl2 in mediating survival of TR9 Ron cells, a well-defined Bcl2 inhibitor, ABT-263, was utilized [25]. Addition of ABT-263 to TR9 Ron cells diminished HGFL-dependent survival to basal levels under detached conditions (Figure 7A). ABT-263 Treatment also reduced the survival of TR9 Ron cells in absence of HGFL, suggesting that Bcl2 is an important survival factor in prostate cancer cells that prevents anoikis even in the absence of Ron activation. STAT3 ablation in TR9 Ron expressing cells was accomplished utilizing lentiviral transduction with a shSTAT3 construct. STAT3 was efficiently knocked-down in the TR9 Ron cells with knockdown correlating with decreased Bcl2 levels and reduced survival under detached conditions (Figure 7B and data not shown).

## DISCUSSION

Several reports have implicated the Ron receptor tyrosine kinase as a driver of tumorigenesis [4, 13, 14, 26, 27]. However, much less is known about the requirement of the Ron ligand, HGFL, in tumorigenesis. Interestingly, while a number of studies clearly show the ability of HGFL to induce Ron activation *in vitro*, there are also studies that have documented ligand-independent effects of Ron [28, 29]. This report is the first to examine the role of endogenous HGFL in the development and progression of cancer and provides genetic evidence that HGFL is necessary for Ron activation and prostate tumorigenesis in the TRAMP model. These results are consistent with recent information demonstrating the coordinate overexpression of HGFL, Ron and Mtsp1 in a set of human breast cancer patients, which correlated with poor patient survival and early death [15]. In addition, ectopic overexpression of HGFL in murine breast cancer cells was able to promote tumor growth and increase the distribution of metastases. Similarly, overexpression of HGFL in human small cell lung carcinoma cell lines also increased the number of micrometastasis in the liver upon intravenous inoculation in SCID mice [15, 16]. While under the time frame of our study metastases were not present to evaluate disseminated tumor growth, our data clearly establish host HGFL as a critical regulator of cancer cell survival and ensuing tumor growth. Further, our current study led to the novel discovery that HGFL protein is upregulated in the tumor proper during tumorigenesis. The upregulation of HGFL in the TRAMP+ model was further supported by a large human prostate cancer data analysis wherein HGFL protein levels were found to be significantly increased in prostate carcinomas compared to minimal levels observed in normal tissue. Collectively, the current data support the concept that the level of HGFL may be an important factor governing tumor growth as well as the extent and survival of metastatic lesions.

We have previously shown that TRAMP+ mice with a loss of Ron receptor signaling also led to the development of smaller prostate tumors [13], similar to the results from the HGFL-/- TRAMP+ mice. While Ron expression was not altered in the HGFL deficient mice, HGFL loss resulted in significantly reduced Ron activity as judged by kinase assays. The similarity in phenotype of the Ron deficient versus HGFL deficient TRAMP+ mice suggests an important ligand dependent relationship in tumor development and progression. In comparing prostate tumor characteristics in HGFL-/- TRAMP+ mice to TRAMP+ mice proficient for HGFL, no differences in cellular proliferation rates were observed and are similar to studies in TRAMP+ mice lacking Ron. In contrast, striking differences were noted in prostate cancer cell survival in wild-type TRAMP+ mice compared to those with defects in Ron signaling. Our current study suggests that the HGFL-dependent pro-survival function of Ron may at least in part be mediated by activation of STAT3 signaling that is also Bcl2-dependent. Similarly, increased activation of STAT3 is a prominent feature in hypoxic regions of ovarian cancers and has been shown to be important for cancer cell survival and proliferation [30]. Treatment of hypoxic ovarian cancer cells with the STAT3 inhibitor, HO-3867, led to rapid decreases in STAT3 phosphorylation and supports the use of novel and specific STAT3 inhibitors as an attractive and viable therapeutic option in conjunction with HGFL/Ron inhibition for prostate cancer patients [30]. Interestingly, recent data has shown that Ron loss in the host microenvironment, and in particular in myeloid cells, was able to reduce prostate cancer cell growth [31, 32]. While our *in vivo* studies cannot differentiate which cell compartment is responsive to HGFL in the promotion of prostate cancer cell growth, our present data are consistent with the increased penetration of F4/80-positive macrophages into the tumor proper following loss of Ron signaling in myeloid cells [31]. Further examination into cell type specific mechanisms necessary for the effect of HGFL is needed to clarify the multiple potential mechanisms that may be operant with this unique ligand-receptor pair.

Prostate tumors from HGFL-/- TRAMP+ mice have reduced tumor vascularization compared to HGFL+/+ TRAMP+ mice. We previously established that prostate tumors from TRAMP+ mice deficient for Ron signaling also exhibit reduced vascularization which was evident by reduced CD31 positive cell immunohistochemistry as well as a 3-fold decrease in VEGF mRNA [13]. Our findings suggest that HGFL-dependent Ron activation and subsequent induction of STAT3 may be partially responsible for the temporal induction of VEGF. VEGF has been shown to be a direct target of STAT3 [33] and our current studies provide novel insight into Ron-mediated vascular development in prostate cancer. Recent studies in mouse mammary tumors have shown that activation of the Ron related receptor, c-Met, is responsible for increased tumor blood flow as well as serving as a mechanism of resistance to a number of targeted therapies including those directed against EGFR, VEGF and B-Raf [34]. A subset of ovarian cancers has also recently been shown to be responsive to c-Met inhibition with targeted trials underway [35]. High levels of c-Met have also been frequently demonstrated in prostate cancer cells [36]. Given that Ron and Met are related and that these receptors have been reported to interact, targeting both Ron and Met may provide an amplified therapeutic effect for prostate cancer patients by reducing tumor blood flow and nutrients to the tumor proper leading to tumor cell death and prolonged patient survival [37, 38].

HGFL has been shown to exert anti-anoikis effects on MDCK cells under detached conditions [39]. Anoikis is a type of cell death where epithelial cells undergo apoptosis due to contact loss with the underlying extracellular matrix [40, 41]. Not surprisingly, cancer cell resistance to anoikis correlates with tumor malignancy [42, 43]. Our report shows, for the first time, that HGFL provides a survival advantage to prostate cancer cells through a Ron dependent pathway and that this effect is associated with the activation of STAT3 and Bcl2. Based on HGFL immunohistochemical staining of both human and mouse prostate cancer specimens, an increase in cancer cell-produced HGFL is consistently observed. An attractive hypothesis is that prostate cancer cells start expressing HGFL to aid in escaping anoikis via HGFL/Ron autocrine signaling, which in turn, increases cancer cell survival. While our current studies provide evidence for increased HGFL/Ron-mediated prostate tumor cell survival, further studies are necessary to delineate the differences between endocrine sources and cancer cell-produced HGFL.

Our prior studies have shown that the Ron receptor tyrosine kinase is a crucial factor in prostate tumorigenesis and our current study facilitates a deeper understanding of HGFL-dependent Ron activation in this process. These findings provide a contemporary hypothesis that prostate cancer cells utilize this pathway to escape cell death. Further studies are needed to elucidate if advanced prostate cancers are dependent on this pathway to evade androgen dependence. Our data suggest that Ron receptor tyrosine kinase function in prostate tumorigenesis is dependent on ligand-mediated activation with these findings implicating for the first time the Ron ligand, HGFL, as a novel therapeutic target in prostate cancer.

## MATERIAL AND METHODS

### Mice

C57Bl/6 TRAMP mice were purchased from The Jackson Laboratory (Bar Harbor, ME, USA). Mice containing a germline deletion of HGFL (HGFL-/-) have been characterized previously [44]. TRAMP+ mice were crossed with HGFL-/- mice to generate HGFL-/- TRAMP+ mice. Only hemizygous TRAMP+ male mice (hereafter referred to as TRAMP+) were used for experimental analyses. All mice used for the experiments were on a pure C57Bl/6 background. The use and maintenance of animals was performed under protocols approved by the Institutional Animals and Use Committee of the University of Cincinnati. For weight measurements, mice were euthanized and the genitourinary (GU) complex (consisting of prostate, seminal vesicles, urethra and empty bladder) was first removed and weighed. The prostate was dissected from the GU complex and either snap frozen in liquid nitrogen or fixed in 10% formalin for further analysis.

### Prostate histology, immunohistochemistry and scoring

Murine prostate histopathology was graded as previously defined [26]. Immunohistochemistry of prostate sections from paraffin-embedded 30-week-old wild-type (HGFL+/+), HGFL-/-, HGFL+/+ TRAMP+ and HGFL-/- TRAMP+ prostates were processed as described previously [4, 14]. For human HGFL staining, the HGFL (N-19) antibody (1:50; Santa Cruz Biotechnology, Santa Cruz, CA, USA) was applied to the 200 Case Grade/Stage human prostate cancer tissue microarray (from the Prostate Cancer Biorepository Network through Bruce J. Trock, Ph.D. at John Hopkins University). For mouse HGFL staining, the HGFL (T-19) antibody (1:100; Santa Cruz Biotechnology, Santa Cruz, CA, USA) was applied to mouse prostate tissues. HGFL staining on human prostate cancer tissue arrays was graded based on the staining intensity (“0” – no staining; “1” – weak staining; “2” – intermediate staining; “3” – strong staining) multiplied by the percentage of positively staining epithelial cells. F4/80 staining was performed as described previously [45]. For the analysis of proliferation, 2 hours before harvest, 30-week-old HGFL+/+ TRAMP+ and HGFL-/- TRAMP+ mice were injected intraperitoneally with BrdU (24mg/kg body weight) and sections from paraffin-embedded prostate tissue were stained utilizing a BrdU staining kit (Amersham, Piscataway, NJ, USA) according to the manufacturer's instructions. Cleaved Caspase-3 staining was performed according to the manufacturer's instructions (1:100; Cell Signaling Technology, Danvers, MA, USA). For TUNEL analyses, prostate sections from 30-week-old mice were stained using an In situ Cell Death Detection Kit (Millipore, Billerica, MA, USA) according to the manufacturer's directions. Positive cells were counted and normalized to the total number of prostate epithelial cells per field and represented as a percentage of total epithelial cells.

### Quantitative real-time PCR (qRT-PCR)

RNA was isolated from frozen prostate tissue from 30-week-old mice using Trizol reagent (Invitrogen, Carlsbad, CA, USA). The high-capacity complementary DNA kit (Applied Biosystems, Foster City, CA, USA) was utilized to convert RNA to cDNA as per manufacturer's instructions, and SYBR green incorporation (Roche Diagnostics) was used to measure gene expression. Primer sequences used were as follows: mouse HGFL forward, GCTGTGGCATCAAAACCT and reverse, TGGAAAGGGTGCGAGT; mouse Ron forward, TCCCATTGCAGGTCTGTGTAGA and reverse, CGGAAGCTGTATCGTTGATGTC. Gene expression values were normalized to mouse β-glucurodinase (GUS) forward, TTGAGAACTGGTATAAGACGCATCAG and reverse, TCTGGTACTCCTCACTGAACATGC. Relative gene expression values are shown.

### Immunoprecipitations, Western blot analyses and kinase assays

For protein analyses, prostate tissues were homogenized in protein lysis buffer [50 mmol/L Tris (pH 7.4), 0.5% Triton X-100, 0.5% IGEPAL, 150mmol/L NaCl, 2mmol/L EDTA] containing protease inhibitor (Complete Mini, EDTA-free, Roche Diagnostics, Indianapolis, IN, USA) and 1 mmol/L Na3VO4. Protein concentrations were determined using the MicroBCA kit (Pierce Biotechnology, Rockford, IL, USA) according to manufacturer's instructions. A total of 100 μg of protein was subjected to western blot analysis as described previously [4]. Primary antibodies used were: anti-Ron C-20 (1:200; Santa Cruz Biotechnology), anti-HGFL (T-19) mouse (1:500; Santa Cruz Biotechnology), total STAT3 (1:1000; Catalog no. 9139, Cell Signaling), pSTAT3 (1:800; Catalog no. 9145, Cell Signaling), pAKT (1:1000; Catalog no. 4060, Cell Signaling), total AKT (1:1000; Catalog no. 4691, Cell Signaling), p-p44/42 MAPK (1:1000; Catalog no. 4370, Cell Signaling), total p44/42 MAPK (1:2000; Catalog no. 9107, Cell Signaling) and actin C4 (1:40,000; received as a gift from Dr. James Lessard at Cincinnati Children's Hospital Medical Center, Cincinnati, OH, USA). Peroxidase-conjugated secondary antibodies were applied, membranes were developed using ECL Plus Western Blotting detection reagent (GE Healthcare, Piscataway, NJ, USA), and protein bands were detected using autoradiography. Kinase assays were performed as previously described [4] with 500 μg of total protein lysate used for immunoprecipitation in 1ml of phosphate buffered saline containing protease inhibitors (Complete Mini, EDTA-free, Roche Diagnostics, Indianapolis, IN, USA) with 2 μg of primary antibody (anti-Ron C-20, Santa Cruz Biotechnology, Santa Cruz, CA, USA) and dephosphorylated myelin basic protein (Millipore, Billerica, MA, USA) as the substrate.

### Cell culture, HGFL overexpression, and cell viability assay

Mouse prostate cancer cells (referred to as TR9 cells) were derived from the prostates of TRAMP+ mice deficient for the Ron tyrosine kinase signaling domain [13]. Cells were cultured in high glucose DMEM media supplemented with 5% FBS and 5% NU-Serum (Becton, Dickinson and Company, Franklin Lakes, NJ, USA) and were maintained at 37°C incubator with 5% CO2. To generate Ron overexpressing cells, TR9 prostate cancer cells were transduced with a pCDH lentiviral vector (System Biosciences, Mountain View, CA, USA) containing Ron complementary DNA. Cells were selected with Puromycin (1μg/ml). To generate cells deficient in STAT3 signaling, TR9 cells overexpressing Ron were transduced using a pLKO lentiviral vector containing a shSTAT3 construct (5'-CCTGAGTTGAATTATCAGCTT-3', TRCN0000071456). 4 × 10^6^ control cells and cells re-expressing Ron (TR9 and TR9 Ron, respectively) were plated in duplicate in serum-free DMEM on Poly(2-hydroxyethyl methacrylate)-coated (Sigma-Aldrich, St Louis MO, USA) tissue culture plates as described previously [46]. Recombinant HGFL (rHGFL) (R&D Systems, Minneapolis, MN USA) was added daily at a concentration of 100ng/ml. Cell suspensions were collected at day 1 and 6, spun at 900rpm for 5 minutes, aspirated and 200ul of 5mg/ml Thiazolyl Blue Tetrazolium Bromide (MTT) (Sigma-Aldrich) in PBS was added for 2 hours at 37°C. Formed formazan crystals were dissolved in 200ul of DMSO and absorbance was taken at the wavelength of 570nm. For Bcl2-dependent survival studies, 0.1um of ABT-263 in DMSO (Santa Cruz Biotechnology) was added at day 1.

### Statistical analysis

Data are expressed as mean ± standard error (SE). Statistical significance comparing experimental groups was determined by using Student's t-test for pairwise comparisons or Analysis of Variance for comparison of multiple groups. Graph Pad Prism software (La Jolla, CA USA) was used for all calculations. Differences between groups were determined as significant when P<0.05.

## SUPPLEMENTARY MATERIAL AND FIGURE


