# Supplementary material for: Hepatocyte growth factor-like protein is required for prostate tumor growth in the TRAMP mouse model
Source: Oncotarget. 2014 Jun 26;5(14):5547–58. doi: 10.18632/oncotarget.2139 (PMC4170603; doi:10.18632/oncotarget.2139)
Supplement: Supplementary file 1 [file oncotarget-05-5547-s001.pdf]

Hepatocyte growth factor-Like protein is required for prostate tumor growth in the TRAMP mouse model

Supplementary Material

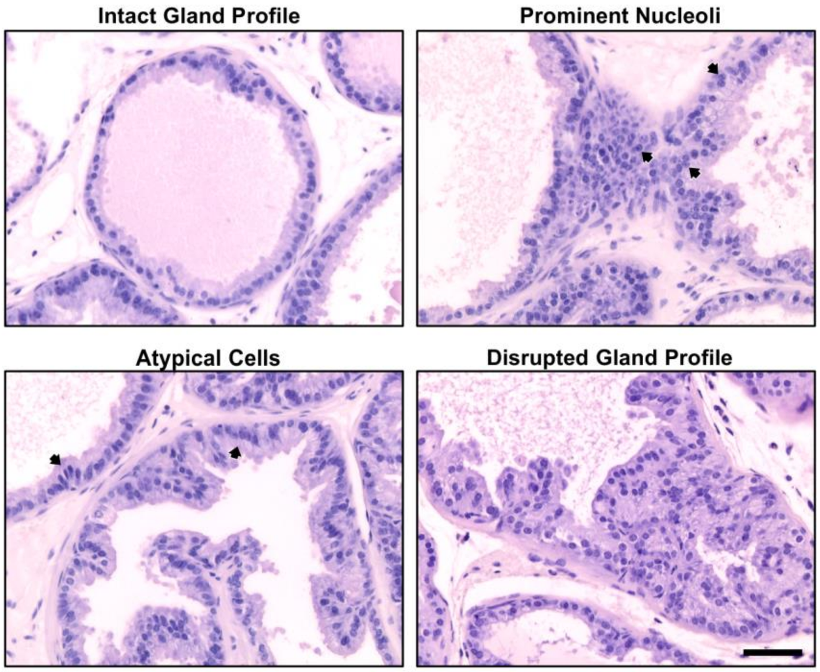

Age/ Genotype	Prominent nuclei	Atypical Cells	Intact Gland Profile	Disrupted Gland Profile
30w HGFL+/+	47%	3%	100%	0%
30w HGFL–/–	91%	84%	0%	63%

Supplementary Figure S1. Histological characterization of prostates from HGFL+/+ and HGFL-/- mice. 30-week old prostates from HGFL+/+ and HGFL-/- mice were evaluated for histologic abnormalities. Ten different fields from three different specimens from HGFL+/+ mice prostates and five different specimens from HGFL-/- prostates were analyzed. Representative images for each histological classification are shown. Scale bar is 50μm. Detailed pathological features observed per genotype are tabulated below. Prostates from HGFL+/+ and HGFL-/- mice were histologically classified by examination for the presence of prominent nuclei, atypical cells and disrupted glandular profiles as previously described (19).
